# Barriers and drivers to adopting a plant-rich Mediterranean diet in a high-income country: A qualitative study

**DOI:** 10.1177/13591053251354851

**Published:** 2025-08-03

**Authors:** Nicole Allenden, Amy D Lykins, Keri L Phillips, Suzanne M. Cosh

**Affiliations:** University of New England, Australia

**Keywords:** behaviour change, behaviour change wheel, meat consumption, Mediterranean diet, sustainability

## Abstract

Eating a plant-rich diet is considered essential for human and planetary health and the Mediterranean diet offers a realistic way to increase this. Gaining greater knowledge of the barriers and drivers to consuming the Mediterranean diet in residents of high-income countries was the aim of the current study. Semi-structured interviews were conducted with 16 adults residing in Australia who ate either an omnivore or plant-rich diet. Using reflexive thematic analysis (RTA) and the behaviour change wheel (BCW), an in-depth exploration of these barriers and drivers was conducted. Key barriers were: (1) changing ingrained meat habits, (2) lack of physical and mental availability, (3) household influences, (4) meat perceived as tasty and Mediterranean diet foods as bland and (5) minimal knowledge of the nutritional benefits of Mediterranean diet foods. Our findings emphasize the need to consider multiple individual and environmental barriers when designing behaviour change interventions to increase Mediterranean diet adoption.

## Introduction

High meat consumption – particularly red and processed meat – is linked to a range of chronic health problems (Demeyer et al., 2016; Li et al., 2018; Micha et al., 2010; Pan et al., 2011), and livestock husbandry produces 16.5%–30% of total global greenhouse gas (GHG) emissions (Twine, 2021), prompting concerns about human *and* planetary health. With meat consumption continuing to rise globally (FAO, 2017), these risks are ever-increasing. To fully understand the scale of this crisis, meat consumption is best viewed through the holistic ‘One Health’ model (Zinsstag et al., 2011). This model emphasizes the inter-connections between human, animal and planetary health, and posits that human health is reliant on the health of our eco-system. High meat consumption clearly threatens human health directly, but also threatens our eco-system, prompting an urgent review of our dietary behaviours. Promoting plant-rich eating is now considered a vital strategy to support human health and a sustainable food system (e.g. low environmental impacts, nutritiously adequate; IPCC, 2022, 2023) and aligns with the ‘One Health’ model. Greater health and environmental gains can be achieved if people in high-income countries adopt plant-rich eating, as their meat consumption, on average, is above expert-recommended targets (Willett et al., 2019). Developing interventions to increase plant-rich eating is therefore vital if we are to support human health and achieve a sustainable food system.

The Mediterranean diet (MD) is a plant-rich diet that has positive health and environmental outcomes (Aleksandrowicz et al., 2016; Yacoub Bach et al., 2023). It is comprised of high levels of plants (fruit, vegetables, whole grains, legumes, and olive oil) and moderate (chicken, fish) to low (red meat) levels of meat (Bach-Faig et al., 2011). It is associated with reduced risk of developing heart disease, type 2 diabetes, dementia and some cancers (Demeyer et al., 2016; Li et al., 2018; Micha et al., 2010; Pan et al., 2011; Shannon et al., 2023), and emits fewer GHG emissions and uses less land and water than an omnivore diet (Aleksandrowicz et al., 2016; Batlle-Bayer et al., 2020). These health and environmental credentials reveal the MD as a worthwhile plant-rich diet to promote, however adoption remains low (Allenden et al., 2022). To that end, exploring the drivers and barriers to people adopting the MD in high-income countries with high occurrence of ‘lifestyle’ diseases is crucial to guide development of interventions to enhance their likelihood of success in changing dietary patterns.

To harness the health benefits of the MD, health researchers have assessed the barriers and drivers of MD adoption in populations at risk of developing health conditions (McEvoy et al., 2018; Moore et al., 2018). The barriers ranged from a lack of knowledge of the MD and negative attitudes towards MD foods (e.g. olive oil, nuts, fish), limited cooking skills for MD dishes, resistance to changing current eating habits, perceived financial cost increase, lack of time and cultural food norms. In a study of Australian adults, Scannell et al. (2020) found that reduced perceived behaviour control (i.e. self-efficacy) had the greatest impact on MD adoption. While insights can be gained from this study, more is clearly needed with community populations to further these findings. Additionally, behaviour change models—considered best practice for developing interventions—are underutilized in this field, with the few models employed (e.g. theory of planned behaviour) not always focussed on habitual behaviours, which are considered essential in food choices (Klöckner, 2013; Moore and Boldero, 2017; Scannell et al., 2020).

**Figure 1.**
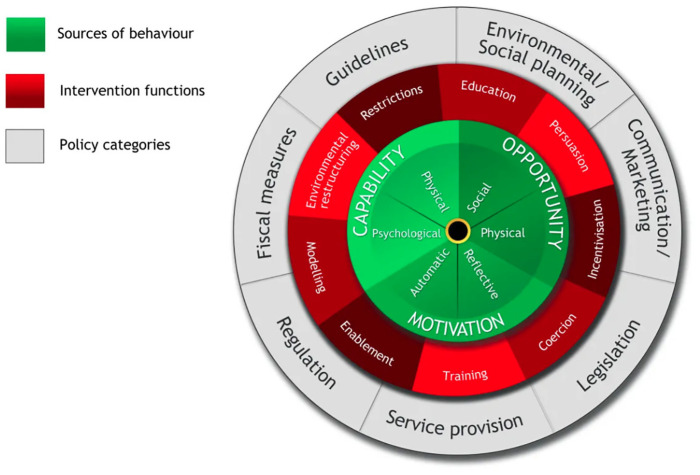
The behaviour change wheel. *Source*: Reprinted from ‘The behaviour change wheel: A new method for characterizing and designing behaviour change interventions’, by Michie et al. (2011), Implementation Science, 6(42), p. 7. Copyright [2011] by Michie et al., licensee BioMed Central Ltd. Used under Creative Commons Attribution Licence: http://creativecommons.org/licences/by/2.0.

The behaviour change wheel (BCW) is a comprehensive behaviour change framework that may overcome this limitation.^1^ Each COM-B component is assessed individually and collectively to understand both individual and environmental factors (Figure 1; Michie et al., 2011)^2^.

The COM-B model has been used previously to promote healthy (Barker et al., 2016; McEvoy et al., 2018) and pro-environmental behaviours (Allison et al., 2021; Sundaraja et al., 2021), and more recently to assess meat reduction behaviours in young people (van Den Berg et al., 2022) and adoption of the MD in people at risk of developing cardiovascular disease (McEvoy et al., 2018). The current study, however, is the first to apply this model to qualitatively explore the adoption of the MD in a community sample. Given the myriad of individual and environmental factors that influence dietary behaviours, the current study aims to gain an in-depth understanding of the barriers and drivers to MD adoption using reflexive thematic analysis (RTA) and COM-B model components, to inform intervention(s) to promote a plant-rich diet that people are willing to consume.

## Material and methods

### Participants

Participants were recruited via first-year psychology students, social media and word of mouth. All participants completed an online survey to determine study eligibility. Inclusion criteria were being aged 18+ years, residing in Australia, living in a household with at least one other person and being the main or joint cook (i.e. to assess participants’ meal and preparation decisions and the possible impacts of other household members). Using purposive sampling (see Kelly, 2010), eligible participants were then selected based on their current diet and likelihood of adopting the MD in the future (see Supplemental Material for more detail). The researchers aimed to interview a broad cross section of participants in each group to obtain a wide range of perspectives on potential barriers and drivers.

From 100 people who completed the screening survey, 30 respondents who met eligibility criteria were invited to take part in interviews. Sixteen interviews were conducted, at which point data saturation was deemed to have been met. Data saturation was determined by the first author by applying a high-order grouping of salient themes as they evolved during the interview process (Hennink and Kaiser, 2022), and as no additional novel information or ideas emerged. All participants received a $50 e-gift voucher for participating in the interview, and university students also received class credit. See Supplemental Data for descriptive and diet information for each participant.

### Procedure

Ethics approval was provided by the University of New England’s Human Research Ethics Committee (Approval No. HE21-014).

#### In-depth interview

Interviews were conducted by the first author via Zoom between March 2021 and January 2022. Informed consent was obtained prior to commencing the interview. The MD, meat-reduction literature (Lacroix and Gifford, 2019; Malek et al., 2019; Stoll-Kleemann and Schmidt, 2017) and COM-B informed the semi-structured interview guide (see Supplemental Material), with questions covering capability, opportunity, and motivation (i.e. dietary habits, dinner decision-making processes, reasons for reducing [or not reducing] meat, and household influences included), with this broad approach also used to capture new insights. General questions about current dinner habits were asked first before MD and meat reduction questions to reduce social desirability bias (Althubaiti, 2016) and gather descriptions of dietary behaviour. Meat (and meat reduction) was a core food (behaviour) in the discussion guide given omnivores need to reduce their meat levels to align with the MD. Participants were shown the MD Pyramid (Medical News Today, n.d.) and a MD plate example (Durrer Schutz et al., 2019; see Supplemental Data) to assess MD awareness. Participants were also asked questions about meat consumption, human health and climate change to assess their awareness of the negative impacts that meat can have on human health and the environment.

Using visual research methods (Pink, 2012), participants moved to the kitchen for a video observation of their pantry/fridge. Two participants were not able to conduct the observation and were asked to describe what they thought was in their pantry/fridge and take photos and send via email, with one participant completing this request. During the observation, participants often discussed their eating and cooking habits in greater detail after receiving visual prompts. Interviews lasted between 1 and 2 hours, with an average length of 1.5 hours. All interviews were video recorded and transcribed verbatim, with pseudonyms applied.

### Analysis

A reflexive thematic analysis (RTA) was used to analyse the data (Braun et al., 2022; Braun and Clarke, 2019) using a combined inductive and deductive approach (Fereday and Muir-Cochrane, 2006). This combined approach matched the aims of the study, as themes could be generated directly from the data (inductive coding) and the COM-B could be integrated into the analysis (deductive coding). Using the RTA approach, the first author became familiar with the data by transcribing and re-reading transcripts. Initial code development was then conducted by identifying and labelling relevant segments of the transcripts using colour coding. Initial codes were refined (e.g. expanded, joined) and modified (e.g. relabelled) by re-reviewing the transcripts and the notes.

Following inductive coding, a deductive approach was then applied by mapping the codes onto the COM-B sources of behaviour: ‘Capability’‘Opportunity’‘Motivation’ and specific factors (e.g. psychological capability – knowledge). Codes with shared meaning (and COM-B factors) were then collated to generate initial themes and subsequent subthemes. Themes and subthemes were re-mapped onto the COM-B and further refined. Several differences were identified between participants consuming a plant-rich diet and those consuming an omnivore diet, although group differences were not detected between the two omnivore groups, *Likely to Adopt* and *Unlikely to Adopt*, therefore, these two groups were joined as *Not Engaged*. Barriers and drivers are discussed under each theme. A comparative analysis was not conducted, though per the qualitative research process (Phoenix and Orr, 2017), participants discussed their unique experiences which were considered in relation to different and similar broad MD experiences across the sample.

Overall, an iterative and reflexive process was undertaken. The influence of the first author’s prior knowledge of the COM-B and meat consumption literature on the analysis was considered. Codes, themes, and subthemes were initially generated by the first author. Multiple reviews and code checks (Miles and Huberman, 1994) were then conducted in collaboration with the second and last author, being psychologists, with the last author less familiar with the COM-B, specifically in the context of plant-rich diets. Inter-rater reliability was not used as this method is not generally viewed as in-line with RTA (Braun and Clarke, 2021). Instead, we used an open review procedure in line with a ‘critical friends’ approach (Smith and McGannon, 2018), which enabled the exploration of multiple and alternative explanations and interpretations as they emerged. This collaborative inductive and deductive process ensured a thorough and rigorous methodological approach that enhanced research outcomes by valuing participant voices and applying a theory driven assessment (Braun et al., 2022; Fereday and Muir-Cochrane, 2006).

## Results

**Figure 2.**
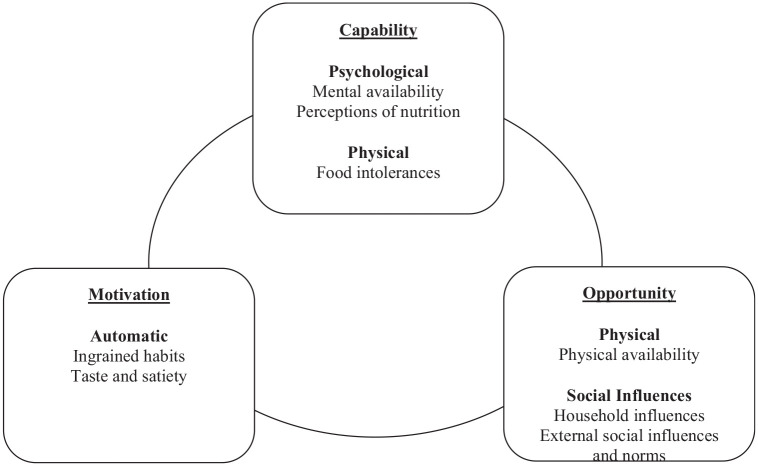
A summary of results for themes organized according to the COM-B model.

Using the overarching COM-B themes of capability, opportunity and motivation, five themes, relating to the adoption of the MD, were generated: (1) ingrained habits (repetition and culture/childhood), (2) perceived lack of time (mental and physical availability), (3) social influences (household and external), (4) taste and satiety, and (5) human health (perceptions of nutrition and food intolerances; see themes according to the COM-B in Figure 2). Barriers to adopting the MD were identified in each theme, with drivers also identified.

### Theme 1: Ingrained habits (automatic motivation)

All participants reported habitual meal planning, often stating that changing these ingrained habits was a key barrier to MD adoption. Two subthemes were identified; these were ‘dinner repetition’ and ‘culture and childhood influences’. Given the repetitive and automatic nature of habitual behaviours, this theme is best characterized by automatic motivation in the COM-B model.

#### Dinner repetition

Participants across all groups cooked the same meals on a weekly or fortnightly basis, with a typical dinner repertoire ranging from 5 to 15 meals. This habitual meal planning was reportedly a barrier to dietary change in multiple ways. Participants reported minimal use of recipes, with most dinner meals being ‘*in my head*’. The same food purchasing, meal choice, meal preparation and consumption behaviours were described as repeated on a regular basis.

*Engaged* and *Not Engaged* participants reported different meat levels in their dinner meals. *Not Engaged* participants frequently mentioned meat first, whereas *Engaged* participants reportedly consumed less or no meat for dinner. Several *Not Engaged* participants mentioned that changing these ingrained meat-focussed habits was hard, representing a key barrier to consuming a MD (‘*…because it*’*s the path of least resistance, we just keep eating what we eat now and not think about it*’, *Ryan, Not Engaged*). However, some *Not Engaged* participants had modified their meal habits to align more with the MD (e.g. reducing red meat, incorporating one meat-free meal in their weekly dinner repertoire), with health and ethical concerns identified as the main drivers, (‘*…the carbon dioxide associated, and the water associated with meat production, and it*’*s a little bit for health reasons*’, *Laura, Not Engaged*).

#### Culture and childhood influences

The majority of *Not Engaged* participants described eating meat-focussed meals in their childhood and mentioned the influence of their culture on these eating behaviours (‘*…very meat and three veg kind of traditional, you know, Anglo cooking*’, *Martin, Not Engaged*). *Not Engaged* participants continued to consume these meat-focussed meals, indicating that early eating experiences influenced current eating habits (‘*I find meat and three veg easy…it came from my childhood I guess*’, *Jessica, Not Engaged*).

*Engaged* participants who identified with non-Australian cultures and/or reported early eating experiences in line with the MD also described the influence of culture and early childhood eating experiences on their current eating habits (‘…*the portion size of the meat, would be really quite small…*[came] *from my childhood*’, *Greta, Engaged*). In contrast, *Engaged* participants who identified as Australian described the challenges associated with changing from a meat-focussed diet to a plant-rich diet, (‘*…most of the meals…I knew how to cook…were meat-based meals so, yeah… definitely made it harder*’, *Justin, Engaged*), with participants starting with small shifts *(*‘*so one* [vegetarian] *meal a week regularly…and I introduced the fish meal probably once weekly as well…and I*’*ve just slowly increased it from there*’, *Leah, Engaged*) to change these ingrained habits.

### Theme 2: Perceived lack of physical and mental availability (physical opportunity; psychological capability)

This key theme related to time. All participants mentioned that work, study and childrearing commitments reduced their physical and mental availability to make dinner. In the context of these commitments, participants often described dinner as ‘*exhausting*’ and a ‘*chore*’, with familiar and convenient meals preferred. For *Not Engaged* participants, changing their current dinner repertoire to align with the MD was described as effortful, with perceived lack of time a key barrier. Two subthemes were identified; these were ‘physical availability’ and ‘mental availability’. Per the COM-B, these subthemes relate to physical opportunity (i.e. less physical availability to make dinner) and psychological capability (i.e. less mental availability for dinner decision-making and less knowledge of MD and meat-free recipes).

#### Physical availability

Most participants stated that their work and childrearing commitments impacted what time they got home, resulting in less physical time to make dinner (‘*We both get home late…therefore* [I] *make a meal i.e. always something very easy to cook*’, *Martin, Not Engaged*). Even when participants worked from home, physical availability continued to impact dinner as the boundaries between work, study and home time were frequently blurred:

*‘…like last night…there is nothing that could have made me want to cook dinner at all… I have really full days. I knew I had more to do into the evening and…don’t have the emotional bandwidth to do anything other than just get some of this, you know churn through the work…’, (Laura, Not Engaged)*

Given these time barriers, all participants prepared quick and easy dinner meals. To save time, participants described and showed a range of convenience foods (e.g. frozen peas, tinned chickpeas, taco kit) during the pantry and fridge observation.

#### Mental availability

These commitments also reduced mental availability, with most participants describing last minute dinner decisions due to attending to other tasks first:

‘*…finish work at five or four thirty, I jump in the car and I go get the baby from day care …if I haven*’*t thought about it, it*’*s really hard, you know, to just snap into that mode. I get home and I just get straight into the kitchen and start*’. *(Rebecca, Not Engaged*)

The mental demands of attending to these other tasks made it hard for participants to decide what to cook for dinner (‘…*should I cook this, I don*’*t really feel like it, oh, we don*’*t have enough of this and, like, just more decision making*’, *Jessica, Not Engaged*) with the ‘*effort of changing*’ often described as a key barrier to adopting the MD. Most *Not Engaged* participants described meat meals as quicker and easier than meat-free meals, (‘*It*’*s so easy to like throw a steak on the fry pan and like dinner*’*s done…I don*’*t want to have to be making a new vegetarian meal every day*’, *Kelly, Not Engaged*), as they had less knowledge of MD and meat-free recipes. Both *Engaged* and *Not Engaged* participants (who had reduced some of their meat consumption) explained that they had saved time and mental effort by modifying current meals (i.e. replacing meat with tofu, plant-based meat alternatives [PBMA], or lentils), rather than learning new recipes.

### Theme 3: Social influences (social opportunity)

The third theme related to social influences, that is, other household members and people outside of the home. All participants were influenced by other household members’ eating habits, with some *Not Engaged* participants describing these influences as a barrier to adopting the MD. Children appeared to be the greatest barrier to adopting the MD, with their parents highlighting their preference for meat and carbohydrates over core MD foods such as vegetables and legumes. All participants were influenced by people outside of the home (e.g. friends, family), however, to a lesser extent than household members. Two subthemes were identified; these were ‘household influences’ and ‘external social influences’. Both subthemes relate to social opportunity in the COM-B model as they are factors outside of the individual.

#### Household influences

Most participants reported eating dinner with other household members, with everyone usually eating the same meal (‘*you know, probably 90% of the time we eat the same thing*’, *Justin, Engaged*). Given household members usually ate the same dinner meal, some *Not Engaged* participants reported that the MD would be difficult to adopt if other household members were not interested (‘*I quite like most of the aspects of the Mediterranean diet…the only issue…I*’*m living in a household that not everybody shares that opinion*’, *Laura, Not Engaged*). Making multiple dinner meals to suit individuals needs was considered inconvenient given the perceived time barriers reported above.

When exploring why other household members may not prefer the MD, *Not Engaged* participants described a dislike of legumes and/or a resistance to reducing meat consumption levels, specifically red meat. One participant increased her beef consumption due to her partner’s cultural background, despite eating a MD in her childhood, (‘*I was never a big red meat eater, my partner*’*s predominant meat of choice is always going to beef, it*’*s just what he grew up on in* [South America]’, *Kelly, Not Engaged*), highlighting the influence of other household members. While household members acted as a barrier to reducing meat to be in line with the MD, some *Not Engaged* participants reported their influences on other household members, (‘…[my partner’s] *definitely cut down* [on red meat] *because the rest of us is not eating as much red meat in general*’, *Laura, Not Engaged*). Thus, household influences can be multidirectional, acting as both a driver and a barrier to meat reduction.

Children also presented as barriers to consuming a MD. Participants who were parents stated that dinner was often determined by what their children would eat. Both *Engaged* and *Not Engaged* participants described their children as ‘*fussy*’ eaters who prefer meat and carbohydrates (‘*…*[my children] *are very interested in having say, pasta or rice or mashed potato…they definitely like things like sausages or, um, chicken…*’, *Greta, Engaged*) with other MD foods, such as vegetables and legumes, less preferred or disliked. Parents also mentioned that their children responded with strong emotions when offered less preferred or less familiar food. Dinner choice was therefore determined by what children will eat *and* how they may respond, (‘*…if they*’*re actually going to eat* [dinner]*…how much push will I get, versus how much energy do I have to enforce this tonight*’, *Rebecca, Not Engaged*). Thus, participants reported cooking meat and carbohydrate-focussed meals as they knew their children would eat them.

#### External social influences

Participants’ eating habits were also influenced by people outside of the household. Several *Not Engaged* participants described interacting with friends and family who ate a similar omnivore diet, with fewer interactions with people who ate a MD or other plant-rich diet. This lack of exposure may act as a barrier to adopting the MD. Moreover, some *Not Engaged* participants described how they changed their eating habits to match the social norm of the group. Laura (*Not Engaged*), for example, ate a plant-rich meal when eating out with her vegetarian and pescatarian friends, but consumed an omnivore diet when eating with her extended family (‘*…to say I*’*m only going to eat a Mediterranean diet, that would actually be quite constraining…*[they] *may kind of find it a bit weird if I was trying to put constraints on them*’), highlighting the challenges associated with deviating from the social eating norms of a peer or family group.

### Theme 4: Taste and satiety (automatic motivation)

Another theme identified was the sensory experiences of eating, specifically taste and satiety (or fullness)—an automatic motivation per the COM-B. Most *Engaged* and *Not Engaged* participants reported that meat was tasty, to some extent, with meat preferences less in line with the MD (e.g. dislike of fish and preference for red meat). Most *Engaged* participants also described meat as tasty, (‘…*mincemeat is done in a way and…put on a skewer and…cooked over a fire which is really delicious*’, *Shahen, Engaged*).

Compared to meat, *Not Engaged* participants often described vegetables and legumes as less appealing on taste and texture, (‘*I could just slap up some more veggies, so why don*’*t I? … well that*’*s boring or it doesn*’*t taste good*’, *Jessica, Not Engaged;*‘*I am bit ambivalent about beans in salad, I don*’*t particularly like the texture*’, *Brian, Not Engaged*). In addition to taste, some *Not Engaged* participants found meat to be filling and vegetables less satisfying from a satiety perspective, (‘*…*[meat-free meals] *might not be quite as satisfying after a meal, and then you need to make another meal before you know it*’, *Ryan, Not Engaged*). Given these perceptions around vegetables and legumes, most *Not Engaged* participants reported that meat was easy to make tasty, (‘*I mean salmon*’*s really tasty, you don*’*t need to do much to it at all, a bit of salt and pepper and you*’*re good*’, *Martin, Not Engaged)* compared to vegetable-based dishes (‘*…like with veggies… what do I do to make this taste good enough*’, *Kelly, Not Engaged*). One way people had overcome these taste barriers was by learning new recipes and having greater exposure to MD foods (‘*…since* [becoming] *vegetarian… I*’*ve been liking veggies more and more… I*’*ve just sort of acquired more of a liking to the taste*’, *Justin, Engaged*).

### Theme 5: Human health (psychological capability; physical capability)

The final barrier related to human health. Two subthemes were identified; these were ‘perceptions of nutrition’ and ‘food intolerances’. Meat was perceived as essential for a healthy diet and not easily replaced by plant protein sources. In contrast, carbohydrates, a core food of the MD, were often perceived as less healthy. Another health factor was allergies because some participants presented with food allergies or intolerances to core MD foods (e.g. gluten and legumes). Per the COM-B, these subthemes relate to psychological capability (i.e. knowledge and awareness of food nutrients) and physical capability (i.e. reduced capability to consume certain foods due to allergies).

#### Perceptions of nutrition

 From a nutrient perspective, most participants described meat as important for health, specifically ‘*protein*’ and ‘*iron*’, with iron perceived as more important for women (‘*I have exceptionally low iron so am anaemic and find that red meat works*’, *Kelly, Not Engaged*). Meat was also described as the easy way to get nutrients, with some *Not Engaged* participants less aware of how to replace meat nutrients in a meal (‘[meat]*…have some really great vitamins and minerals that you*’*d have to eat so much veggies to get that kind of thing…I also don*’*t have enough information to make a really informed decision about it. It*’*s probably just a lot of that upbringing*’, *Katherine, Not Engaged*), with this lack of nutrient knowledge a barrier. In contrast, some *Not Engaged* participants reported knowledge of nutritional ways to substitute meat protein, such as legumes (‘*…*’*cause I mean, I know there*’*s other things you can eat that*’*s not meat, ah, for protein, but are* [the children] *gonna eat it… probably not*’, *Rebecca, Not Engaged*), with other barriers, such as household influences, impacting consumption. Further to substituting legumes for meat, some *Engaged* participants ate PBMA as a substitute; however, both *Engaged* and *Not Engaged* participants questioned the health impacts of consuming these ‘*processed*’ foods.

Participants also varied in their perceptions of carbohydrates, another typical MD food nutrient. Several *Not Engaged* participants described carbohydrates as unhealthy, with some adopting a ‘*low-carb*’ diet for health reasons (i.e. high blood pressure, diabetes). Gender differences were also observed, with several *Not Engaged* female participants stating that they reduced their carbohydrate consumption for health reasons (‘*my husband and my daughter eat more of the carbohydrates than I do… just because I*’*m trying to watch what I eat… for health reasons*’, *Katherine, Not Engaged)* and due to messages received from the media (‘*I guess there*’*s a lot of…media out there that say carbs are bad for you*’, *Rebecca, Not Engaged)* on this food type. This had been Rebecca’s own perceptions until she received nutrition education - where whole grain carbohydrates were recommended - when she developed gestational diabetes during her pregnancy. Overall, most *Engaged* participants were aware of the nutritious components of different foods including meat substitutes and carbohydrates. In contrast, nutrient knowledge and awareness was more varied in the *Not Engaged* group, and this posed a barrier to adopting the MD.

#### Food intolerances

Although less common, some participants were simply allergic or intolerant to foods that form part of the MD, namely gluten and legumes, acting as the main barrier to adoption. When shown the MD pyramid, Martin (*Not Engaged*), who described himself as coeliac and intolerant to legumes, highlighted that, (‘*what really trips up with the Mediterranean is yeah, pretty much that bottom layer* [grains and legumes], *I can only have limited amounts of* [those foods]’). Clearly, these intolerances limit Martin’s physical capability to consume these foods and meals and are therefore a barrier to consuming the MD.

## Discussion

The current study aimed to explore the barriers and drivers to adopting the MD by understanding factors that influence a participant’s capability, opportunity and motivation. Results highlighted several key themes across the COM-B model that influenced MD adoption including ingrained eating habits, perceived lack of time, household influences, external social influences, taste and satiety and health (perceptions of nutrition and food intolerances). Both barriers and drivers were identified within the themes, though barriers were more commonly described by participants and consequently formed more of our analysis, likely due to most participants being omnivore eaters. Inter-relationships between each of the themes and COM-B factors were found, indicating that multiple factors need to be considered when developing interventions aimed at increasing the uptake of the MD.

Omnivore participants regularly ate dinner meals focussed on meat, with this automatic motivation impeding their adoption of the MD. Consistent with past research in non-Mediterranean regions (McEvoy et al., 2018), most participants ate more meat, specifically red meat, for dinner than the MD and fewer MD foods (e.g. legumes) overall. Given most participants grew up in Australia and ate similar dinner meals in their childhood, these meat-focussed eating habits were well established, as often found in high-income Western countries (Malek et al., 2019). Several omnivore participants emphasized that the unconscious day-to-day routine of eating meat-focussed dinner meals made change hard, even when they were highly motivated to consume the MD for health and ethical reasons. Given these findings, *habit strength* needs to be considered when designing MD interventions (Rees et al., 2018; Van’t Riet et al., 2011). Our study highlighted that to change eating habits, all stages of the dinner process need to be considered, including habitual dinner decision-making and purchasing practices (Rees et al., 2018).

Insights from participants who had changed their high meat-eating habits emphasized the importance of small dietary shifts, ranging from adding one weekly meat-free meal to swapping red meat for white meat. Participants may build new MD eating habits using action planning where they identify how (meal type) and when (day) they complete the new eating behaviour (Lacroix and Gifford, 2020). Repetition, particularly when performed in the same environment, helps form and maintain new habits (Lally et al., 2011) and self-monitoring techniques (e.g. calendar, app, text messages) can assist people to stay on track with their action plan (Carfora et al., 2019; Rees et al., 2018). Like our participants, these strategies may be more successful with people already motivated to adopt the MD for ethical and health reasons. Further, dietary change also needs to be supported by policies aimed at creating sustainable eating environments (Mozaffarian et al., 2018), in addition to the promotion of individual behaviour change.

Another important automatic motivation identified was taste and satiety or fullness. All participants spontaneously discussed the sensory experience of eating, often describing meat as tasty and MD foods (e.g. legumes, vegetables) as less appealing. This finding is consistent with research in other Western countries where taste preferences are skewed towards meat and vegetarian meals are considered bland (Lea and Worsley, 2001). These taste preferences may impede the adoption of the MD and need to be considered when developing interventions, though some of these taste barriers may be reduced by meat being a component of the MD (Corrin and Papadopoulos, 2017; Graça et al., 2015). Several omnivore participants were less familiar with legumes and questioned their taste and texture, indicating that a lack of exposure had formed negative sensory cues (Rees et al., 2018; Timlin et al., 2020).

To overcome these taste barriers, several participants used recipes to learn how to make tasty plant-focussed meals, which indicates that education and training may be useful interventions (McEvoy et al., 2018). Using existing familiar food experiences (savoury, flavoursome, and satisfying) can also help initiate and sustain new eating habits by activating parts of the brain that signify how a food is likely to taste and how rewarding it may be (de Boer and Aiking, 2017). Likewise, showing visually appealing pictures of MD meals can build new sensory cues that are perceived as rewarding (Michie et al., 2011; Simmons et al., 2005). Making tasty and visually rewarding MD meals also increases the chances of the meal being repeatedly cooked, supporting the formation of new eating habits.

Lack of time was another key theme influencing physical opportunity and psychological capability. Lack of time is a common barrier to dietary change, with people engaging in more habitual responses when stressed (Schwabe and Wolf, 2010). Changing to a MD requires conscious goal-directed intentions (Carfora et al., 2017). In the context of already limited physical time and decision-making capabilities, it is not surprising that omnivore participants perceived MD and meat-free meals as inconvenient (Malek et al., 2019). The increased availability of pre-prepared or ready meals (e.g. frozen, chilled, shelf-stable) in supermarkets (Wooldridge et al., 2021) indicates that many people seek convenient food, and this was prominent among our participants. Educating people on how to use convenient MD foods (e.g. tinned lentils) combined with quick MD recipes may help overcome these time barriers. Small shifts may also help reduce the physical and mental demands of changing dinner meals (Lacroix and Gifford, 2020), with some of our participants overcoming these time barriers by adding meat replacements (e.g. tofu, PBMA) to familiar meals.

Household members also impacted participants’ social opportunity to adopt the MD, with a lack of support from family members a key barrier (Kretowicz et al., 2018). Past research in Australia found that 68% of families were too busy to find meals that the whole household would enjoy, with over half of parents indicating that their dependent children were the most difficult to cook for (McCrindle, 2017). While not specific to the MD, these findings are consistent with our study where children were mentioned as a key barrier, often responding negatively to MD foods (e.g. legumes). As a result, these foods did not become habitual as they were not offered again (Moore and Boldero, 2017). Despite the influence of household structure on eating, this area has received limited attention in relation to meat reduction (Kretowicz et al., 2018; Stoll-Kleemann and Schmidt, 2017). Given our findings, household influences need to be considered, with *environmental restructuring* and *enablement* being common intervention strategies used to target social opportunity (Michie et al., 2011).

The final theme related to psychological capability as participants’ nutrient knowledge could be a driver or a barrier. Several omnivore participants considered meat an essential component of a healthy diet, with some having less knowledge of the nutritional benefits of legumes and whole grain foods. In fact, participants were divided on the health benefits of carbohydrates, with several participants adopting a ‘low-carb’ diet. Participants already eating a plant-rich diet generally had greater knowledge of the associated health benefits of consuming legumes and whole grain foods and the health risks of meat overconsumption (Lea et al., 2006). These findings correspond to nutrition literacy research, with higher literacy levels found to predict healthy eating patterns, including greater adherence to MD foods (Taylor et al., 2019). Education is therefore a possible intervention, though this needs to be considered in the context of the habitual and routine nature of eating rather than a stand-alone strategy (Van’t Riet et al., 2011). Furthermore, several omnivore participants who had high nutrient knowledge mentioned that other household members were the greatest barrier, emphasizing the importance of developing tailored interventions. Nevertheless, health can be a strong driver for dietary change including meat reduction (Penny et al., 2015), so promoting the nutritional benefits of MD foods (and the health risks associated with red meat) can support adoption through increased knowledge. However, such approaches need to consider broader family influences and may incorporate education of the entire family unit. Additionally, MD adoption would extend to greater health benefits (i.e. reduced stroke and cardiovascular disease; Micha et al., 2010) and consequently, reduced healthcare costs (Australian Institute of Health and Welfare, 2024).

### Implications

To our knowledge, this is the first study to qualitatively assess the adoption of the MD using in-depth interviews in a community sample. This contrasts with past research that has mostly focussed on at-risk participants where health conditions required diet change and thus acted as a major driver for behaviour change (Davis et al., 2015; McEvoy et al., 2018; Zacharia et al., 2020). Furthermore, our findings highlighted that household influences, an often-overlooked environmental factor, was a major barrier for many of our participants and needs to be considered when designing interventions.

An advantage of the current study is its naturalistic generalizability; that is, where the results resonate with one’s personal experience (i.e. barriers and drivers to MD adoption for omnivore eaters, in an Australian community sample; Smith, 2018). Given the limited MD research (including interventions) in the Australian (and other high-income countries) community, future research would benefit from assessing these findings with a larger sample using quantitative research measures. Due to this limited research, the current study took a broad approach to understanding barriers and drivers to MD adoption; thus, future research could assess specific MD behaviours (e.g. red meat consumption) individually, incorporating frameworks that specify behaviours (e.g. AACTT; Presseau et al., 2019).

Nevertheless, our study provides vital data to inform the design and evaluation of a community-focussed MD adoption intervention, which are limited in non-Mediterranean and high-income countries, using the intervention functions that form the second layer of the COM-B. For example, to change ingrained meat eating habits and support MD habit formation, training could be used to teach people how to make an ‘easy’ Mediterranean dinner meal that they schedule in once a week (on the same day) for multiple weeks. This intervention could be trialled with specific groups, such as households with children and/or couples with no children and modified for the general population. To then support wide-spread MD adoption in Australia, the third layer of the COM-B (policy categories) can inform marketing campaigns and policy-level initiatives.

### Limitations

A range of recruitment methods were employed in this study; however, people more interested in the MD and plant-rich diets may have been more motivated to participate. Offering incentives in the form of money and course credits (for university students) contributed to the inclusion of a range of eating patterns. University students were also from a regional university that is known for having varied adult age groups, further adding to the inclusion of varied diet experiences and attitudes towards plant-rich diets. Another possible limitation is that a small number of participants were mostly confined to their homes for the interview due to COVID-19-related restrictions. This may have impacted their current eating habits (i.e. fewer dinner meals at restaurants and external social engagements), and in these instances participants discussed their past eating behaviours. A benefit was the collection of additional information about how dinner was impacted by people working from home, being a more common behaviour post-COVID (Parker et al., 2022).

### Conclusion

People in high-income countries need to reduce their meat consumption to support a healthy life and a healthy planet. The MD is a realistic plant-rich diet that can be promoted to high meat eaters to achieve these goals. Our analysis provides key information on the barriers and drivers to consuming the MD in a context where meat consumption is high. In order of priority, key barriers related to: (1) ingrained meat habits, (2) lack of time to change dietary patterns, (3) meat being perceived as tasty, (4) influences of household members, and (5) lack of nutrient knowledge of MD foods, though a tailored approach is recommended given that multiple barriers are often present. Our current food system is unsustainable and practical solutions are needed urgently. The current study provides vital information about how to implement these solutions.

## Supplemental Material

sj-docx-1-hpq-10.1177_13591053251354851 – Supplemental material for Barriers and drivers to adopting a plant-rich Mediterranean diet in a high-income country: A qualitative study Supplemental material, sj-docx-1-hpq-10.1177_13591053251354851 for Barriers and drivers to adopting a plant-rich Mediterranean diet in a high-income country: A qualitative study by Nicole Allenden, Amy D Lykins, Keri L Phillips and Suzanne M. Cosh in Journal of Health Psychology

sj-docx-2-hpq-10.1177_13591053251354851 – Supplemental material for Barriers and drivers to adopting a plant-rich Mediterranean diet in a high-income country: A qualitative study Supplemental material, sj-docx-2-hpq-10.1177_13591053251354851 for Barriers and drivers to adopting a plant-rich Mediterranean diet in a high-income country: A qualitative study by Nicole Allenden, Amy D Lykins, Keri L Phillips and Suzanne M. Cosh in Journal of Health Psychology

sj-docx-3-hpq-10.1177_13591053251354851 – Supplemental material for Barriers and drivers to adopting a plant-rich Mediterranean diet in a high-income country: A qualitative study Supplemental material, sj-docx-3-hpq-10.1177_13591053251354851 for Barriers and drivers to adopting a plant-rich Mediterranean diet in a high-income country: A qualitative study by Nicole Allenden, Amy D Lykins, Keri L Phillips and Suzanne M. Cosh in Journal of Health Psychology

## Notes

1. Behaviour change wheel (BCW).

2. The first layer of the BCW is the capability, opportunity and motivation model of behaviour, otherwise known as COM-B. ‘Capability’ relates to psychological skills (e.g. knowledge, attention) and physical skills to perform the behaviour. ‘Opportunity’ includes influences from both the physical environment, such as available resources and the social environment, such as friends and family. ‘Motivation’ can be either reflective (e.g. intentions, beliefs) or automatic (e.g. habits, emotions).
