# Supplementary material for: Barriers and drivers to adopting a plant-rich Mediterranean diet in a high-income country: A qualitative study
Source: J Health Psychol. 2025 Aug 3;31(4):1345–61. doi: 10.1177/13591053251354851 (PMC12960785; doi:10.1177/13591053251354851)
Supplement: sj-docx-1-hpq-10.1177_13591053251354851 – Supplemental material for Barriers and drivers to adopting a plant-rich Mediterranean diet in a high-income country: A qualitative study [file sj-docx-1-hpq-10.1177_13591053251354851.docx]

**Zoom Semi-structured Interview – Interested Participants**

| **PART 1: EXPLORING DINNER HABITS (30 Minutes)** | |
| --- | --- |
| **Aim** | Explore and understand dinner habits using the Behaviour Change Wheel (COM-B) |
| **Stimulus** | None |

| **DINNER HABITS** | **COM-B & Observation Notes** |
| --- | --- |
| Tell me what meals you usually have for dinner in a general week  **Code frame below (do not prompt). Prompt by saying ‘what else, what else etc.)**  Steak and salad or vegetables  Fish and salad or vegetables  Chicken and salad or vegetables  Pork chop and salad or vegetables  Lamb chop and salad or vegetables  Sausages and salad or vegetables  Bolognaise (Pasta)  Lasagne | **PHYSICAL CAPABILITY: COOKING SKILLS**  **AUTOMATIC MOTIVATION: HABITS, TASTE, EMOTIONS**   - Meal repertoire (unprompted) - Record meat language - Consider where meat sits within decision making hierarchy? (e.g., **steak and vegetables** = meat is the main ingredient and dinner decisions are made around steak).   **PHYSICAL CAPABILITY: COOKING SKILLS, FINANCIAL**  **PSYCHOLOGICAL CAPABILITY: DECISION MAKING – What to cook**  **REFLECTIVE MOTIVATION: PERCEIVED BEHAVIOUR CONTROL (CAPABILITIES), VALUES, INTENTIONS**  **AUTOMATIC MOTIVATION: HABITS, TASTE, EMOTIONS**  **PHYSICAL OPPORTUNITY: CONVENIENCE, HOUSEHOLD CONTEXT/ENVIRONMENT (e.g., cooking for others, availability of food)** |
| Pizza    Stir fry _________(meat)  Noodles_________(meat)  Curry___________(meat)  Any vegetarian meal (e.g., no meat)  Other_____________________________________  Other_____________________________________  Other_____________________________________   - Does everyone eat the same meal? If no, what are the differences? (HOUSEHOLD CONTEXT) - Does everyone eat at the same time? If no, what are the differences? (HOUSEHOLD CONTEXT) - How do you decide what to have for dinner?   *Prompt on (as needed):*   - When do you decide what you will eat for dinner? - Who decides what to cook / eat for dinner? - What do you like about making dinner? - What do you dislike about making dinner? - Who usually cooks dinner? - Do other people in the house cook (when?) - Do the children help you cook dinner (if applicable)? - Do you ever use a recipe when cooking dinner? - Do you like to try new foods/food from different countries? - If yes, which types? - What about the children (if applicable)? - Does anyone have any special food requirements / allergies? How does this affect the meals you choose? |  |
| - What time do you usually eat dinner? - Where do you usually eat dinner (e.g., table, front of TV) - Where do you get your food (own garden, Woolworth, Coles, IGA, Farmer Markets, Pre-made delivery e.g., weight watchers, light and easy - mixture) - Who does the grocery shopping? - How often do you shop for food? - What type of food do you spend the most money on? - Do you get take-away or eat out? If yes, how often, where, and what do you eat? - Are you happy with the food you eat for dinner – would you like to change anything about dinnertime (food, waste, where, when) |  |
| - When you think of meat – what words come to mind?   Over the last week/ which of the following meats have you eaten, and how many times (if applicable)? Is this a typical week?   - Beef - Poultry - Fish/Seafood - Pork - Lamb - Meat-free main meals (specify)   Prompt on why **(i.e., cost, habit, taste, children, convenience, skill)** they eat that meat and type:   - Cost - Taste - It’s what other household members (e.g., children like/will eat) - It’s easy/quick to cook (convenience) - I know how to cook it (skill level) - Organic, Free-Range, No Added Hormones,   Antibiotic-Free, Raised/produced in Australia, Raised/produced by local livestock farmers (variation on Malek et al., 2019)   - Are there any meats you could do without? - What dinner meals did you eat growing up? Did you grow up eating meat – how much? (adaptation of TEMS) | **AUTOMATIC MOTIVATION: HABITS, TASTE**  **REFLECTIVE MOTIVATION: VALUES, INTENTIONS**  **PHYSICAL CAPABILITY: FINANCIAL, SKILL**  **PSYCHOLOGICAL CAPABILITY: KNOWLEDGE**  **PHYSICAL OPPORTUNITY: CONVENIENCE, FOOD ENVIRONMENT** |
| **PANTRY & FRIDGE - BEHAVIOUR**  Can you show me what is in your fridge and freezer?  Talk me through what food you have in there.  PROMPT IF NECESSARY  What meat do you have in there?  What vegetables?  Can you show me what’s in your pantry; again talk me through what’s in there. | **PHYSICAL CAPABILITY: SKILLS, FOOD TECHNOLOGY**  **AUTOMATIC MOTIVATION: HABITS**  **PHYSICAL OPPORTUNITY: FOOD ENVIRONMENT**  *Meat / Eggs / Dairy (note: organic / free range)*  *____________________________________________________________________*  *Vegetables*  *____________________________________________________________________*  *Packaged food (including plant based – veggie burgers etc)*  *____________________________________________________________________*  *Record any other foods that are aligned to a Mediterranean diet (olive oil, nuts)*  *Meat / Eggs / Dairy (note: organic / free range)*  *____________________________________________________________________*  *Vegetables (tinned)*  *____________________________________________________________________*  *Packaged food (e.g., pasta)*  *____________________________________________________________________*  *Record any other foods that are aligned to a Mediterranean and/or Vegetarian diet (olive oil, nuts)* |
| What cooking equipment do you usually use? (equipment i.e., frying pan, microwave, slow cooker, pressure cooker, oven) | **PHYSICAL CAPABILITY: SKILL**  **PHYSICAL OPPORTUNITY: KITCHEN ENVIRONMENT** |

| **PART 2: EXPLORE DINNER DECISION MAKING PROCESS - DESCRIBE A RECENT DINNER MEAL (TONIGHT & LAST NIGHT) 30 Minutes** | |
| --- | --- |
| **Aim** | Explore and understand drivers and barriers per the COM-B, in the context of a recent dinner events to facilitate memory recall |
| **Stimulus** | None |

| **Tonight and last nights’ meal** | **COM-B & Observation Notes** |
| --- | --- |
| **Tonight’s meal**  ***Decision making:***   - What are you cooking tonight? (check if decision has been made) - What made you decide to cook this meal/these meals? - When did you decide to cook this meal/these meals? | **PHYSICAL CAPABILITY: SKILL, FINANCIAL**  **PHYSICAL OPPORTUNITY: CONVENIENCE, HOUSEHOLD MEMBER COMMITMENTS**  **PSYCHOLOGICAL CAPABILITY: DECISION MAKING**  *Record language used* |
| **Last nights’ meal**   - What did you cook last night   **PROMPT IF NECESSARY:**  ***Decision making:***   - What made you decide to cook that meal?   ***Ingredients / Equipment***   - What is in the meal and why did you decide on those ingredients?   Are **meat, vegetables, and processed foods** in the meal?  *Prompt on:*   - Reasons for choosing those ingredients - Taste (how do the ingredients affect the taste?) - Health (how do the ingredients impact health) - Ease of using ingredient - Affordability - Availability of ingredients in freezer, pantry fridge, supermarket   ***Preparation/ Cooking process***   - How long did it take to cook - Was it easy/hard to cook - What cooking equipment did you use? - Did anyone help you with cooking? - Portion sizes they are using (e.g., meat, vegetables) - Why that size? - Is that optimal for you and your family? - What is stopping you from using more or less?   ***Eating***   - Where did you eat dinner? - Did everyone eat the same? - Did everyone eat at the same time? - Taste / sensory experience - What do people eat first/last (including children)   ***Food waste***   - Did everyone eat everything on their plate? - What was left (if any)? What did you do with the leftovers? | **PHYSICAL CAPABILITY: SKILLS, FINANCIAL**  **PHYSICAL OPPORTUNITY: CONVENIENCE, HOUSEHOLD MEMBER COMMITMENTS, FOOD ENVIRONMENT**  **AUTOMATIC MOTIVATION: HABITS, TASTE, EMOTIONS**  *Record language used, and non-verbals.*  * Consider ‘scratch’ cooking and where are they getting ‘help’ (i.e., pre-packaged sauces, frozen / tinned food) |

| **PART 3: UNDERSTAND DRIVERS AND BARRIERS FOR PEOPLE INTERESTED IN MEAT REDUCTION BEHAVIOUR (MEDITERRANEAN / VEGETARIAN DIETS) (15 MINUTES)** | |
| --- | --- |
| **Aim** | To understand the drivers and barriers to engaging in meat reduction behaviours for people who are **interested** |
| **Stimulus** | None |

| **QUESTION** | **COM-B Section & Observation notes** |
| --- | --- |
| You said when I first contacted you that you were open to the idea of (or likely to reduce) reducing some of your meat consumption in the future. Is that correct? | **PSYCHOLOGICAL CAPABILITY: KNOWLEDGE**  **REFLECTIVE MOTIVATION: VALUES, INTENTIONS, SELF-IDENTITY**  **AUTOMATIC MOTIVATION: HABITS, TASTE, EMOTIONS**  **PHYSICAL CAPABILITY: SKILLS, FINANCIAL**  **PHYSICAL OPPORTUNITY: HOUSEHOLD MEMBER COMMITMENTS**  **SOCIAL OPPORUNITY: SOCIAL NORMS/SUPPORT**  **Record their reasons unprompted, record order reasons recalled.* |
| Tell me the reasons why you want to do this?  **PROMPT on the following if necessary:**   - Health reasons - What specifically? - For them, for others? - Environmental reasons - What specifically? - Where did you see or hear that? - What specific messages made you change? - For them, for others? - Animal welfare reasons? - What specifically? - For them, for others? |  |
| - How you previously tried to reduce your meat, what have you specifically done? - What has been easy? - What has been hard? - What is stopping you from reducing your meat consumption? - Do most people you know eat meat/omnivore diet? (Prompt: family, friends, childhood) - Do you know people who eat a meat reduced diet (Vegetarian / Mediterranean diet?) - What do you think of people who eat a Mediterranean and/or Vegetarian diet? - Would important people in your life be supportive of you eating a Mediterranean or Vegetarian diet? (If applicable) - Household Context: Does everyone in the household want to eat a Mediterranean and/or Vegetarian diet? What does that mean for you (prompt on: if you want to eat vegetarian, both vegetarian and non-vegetarian meals must be prepared). |  |
| Take first barrier they mention:  You first mentioned __________is stopping you from changing. How would you overcome that? |  |
| Take second barrier they mention:  You then mentioned __________is stopping you from changing. How would you overcome that? |  |

| **PART 4: EXPLORE REACTIONS TO MEDITERRANEAN/VEGETARIAN DIET (10 - 15 MINUTES)** | |
| --- | --- |
| **Aim** | To provide information about the MEDITERRANEAN / VEGETARIAN diet and assess thoughts, feelings, reactions, and intervention hypotheses to participants who are interested |
| **Stimulus** | Show MEDITERRANEAN and VEGETARIAN DIET DESCRIPTION/VISUAL, MEDITERRANEAN PYRAMID AND PLATE PORTIONS – See PowerPoint |

I’ve shown you the Mediterranean and Vegetarian diet when I first asked if you wanted to participate in this research.

Here it is again and how it differs to the Omnivore diet (**SHOW STIMULUS)**

| **RESPONSE TO THE MEDITTERANEAN/ VEGETARIAN DIET** | **COM-B Section & Observation notes** |
| --- | --- |
| On a scale from 0 – 10, you scored X on how appealing is the Mediterranean (Vegetarian) diet for you personally.   - Why do you say that?   On a scale from 0 – 10, you scored X on how appealing you thought the Mediterranean (Vegetarian) diet would be for your household to follow.   - Why do you say that?   On a scale from 0 – 10, you scored X on how likely are you to follow a Mediterranean (Vegetarian) diet in the future   - Why do you say that? - What stops you from taking up this diet? - What else? - Anything else? | *Get scores from online survey conducted in the recruitment phase.* |
| **Have you heard much about meat and health?***  **Yes:** What have you heard and what are your thoughts?  **No:** Provide information e.g.,  “The overconsumption of red and processed meat has been linked to non-communicable diseases, specifically cardiovascular diseases, some cancers, and type 2 diabetes.” What do you think about that information?  “A diet high in plant-based food and low in meat (particularly red and processed meat), for example, the traditional Mediterranean diets, has been associated with reduced heart failure, stroke, total mortality, cognitive decline, and some cancers” What do you think about that information?  **Have you heard much at about meat and the environment/climate change?***  **Yes:** What have you heard and what are your thoughts?  **No:** Provide information e.g.,  “Livestock contributes to 14.5% of global GHGEs, making livestock production the third largest emitter, following the energy and transport sectors” What do you think about that information?  “Significant environmental benefit comes from changing consumer diets to include fewer animal-based products including the Mediterranean and vegetarian diets” What do you think about that information? | **PSYCHOLOGICAL CAPABILITY: KNOWLEDGE**  **REFLECTIVE MOTIVATION: VALUES, INTENTIONS, SELF-IDENTITY**  **AUTOMATIC MOTIVATION: HABITS, TASTE, EMOTIONS** |
| I’m going to run a few ideas past you about how people could reduce their meat consumption and get closer to the Mediterranean/vegetarian diet. For each, tell me how appealing (a little, moderately, a lot) they are?   - Meat Free Monday - No meat for lunch - Half the serving of meat in every meal - Fill up with carbs - A tin of beans instead of mince - Better (organic) but less meat | **PSYCHOLOGICAL CAPABILITY: KNOWLEDGE**  **REFLECTIVE MOTIVATION: VALUES, INTENTIONS, SELF-IDENTITY**  **AUTOMATIC MOTIVATION: HABITS, TASTE, EMOTIONS**  **PHYSICAL CAPABILITY: SKILLS, FINANCIAL** |

| **PART 5: CO-CREATION OF A MEDITERRANEAN/VEGETARIAN DIET (10 MINUTES) – PARTICIPATORY COLLABORATION** | |
| --- | --- |
| **Aim** | Assess the meals of participants who are interested and compare with the MEDITERRANEAN and VEGETARIAN diets |
| **Stimulus** | Show MEDITERRANEAN/VEGETARIAN Definitions, MEDITERRANEAN PYRAMID AND PLATE PORTIONS – See PowerPoint |

| **ASSESSMENT OF THEIR DIET TO MED/ VEGETARIAN DIET** | **COM-B Section & Observation notes** |
| --- | --- |
| **GO BACK TO THE MEALS THEY HAVE MADE OVER THE PAST FEW DAYS**  **ASSESS THEIR DIET VS THE MEDITERRANEAN and VEGETARIAN DIETS**  Think of the meals you have eaten the last few days – breakfast, lunch and dinner.  **GET THEM TO WRITE DOWN THE MEALS – AS MANY AS THEY CAN REMEMBER**  **Overall diet**  You have your list of meals that you have eaten over the past few days there. How do the meals you have eaten match with the Mediterranean / vegetarian diets?   - What’s different? - What’s the same? - If you had to shift, what would you change? | **PSYCHOLOGICAL CAPABILITY: PROCEDURAL KNOWLEDGE**  **REFLECTIVE MOTIVATION: VALUES, INTENTIONS, SELF-IDENTITY**  **AUTOMATIC MOTIVATION: HABITS, TASTE, EMOTIONS**  **PHYSICAL CAPABILITY: SKILLS, FINANCIAL**  **PHYSICAL OPPORTUNITY: CONVENIENCE, HOUSEHOLD MEMBER COMMITMENTS, FOOD ENVIRONMENT** |
| *Prompts (as required)*  **Portion size:**   - How does the portion size of your meat differ to these guidelines? - How would you change that to be in line with the guidelines? - What difficulties would you have?   **Red Meat:**   - Have you eaten more or less red meat than the guidelines suggest? - How would you change that? - What difficulties would you have?   **Vegetables:**   - Have you eaten fewer servings of vegetables than the guidelines suggest? - How would you change that? - What difficulties would you have doing that?   **Dairy:**   - Have you eaten more or less dairy than the guidelines suggest? - How would you change that? - What difficulties would you have doing that? |  |

| **PART 6**: **MENU (10 MINUTES)** | |
| --- | --- |
| **Aim** | Show a menu of potential MEDITERRANEAN and VEGETARIAN meals to assess reactions (ALL Participants) |
| **Stimulus** | PowerPoint Slide with pictures of MEDITERRANEAN and VEGETARIAN MEALS |

| **RESPONSE TO THE MEDITERRANEAN/VEGETARIAN DIET** | **COM-B Section & Observation notes** |
| --- | --- |
| Go through each meal (what is in it) and record reactions:  PROMPT on if necessary:   - Visual senses, taste, satiety, emotions - Skill level required - Ingredients / availability - Affordability - What’s good / not good - Household context – would others eat it? - Social norms   **See additional questions/prompts below (as required)**  **ASK ABOUT OTHER MEAL IDEAS – consider cultural context – as collaborative approach** | **PSYCHOLOGICAL CAPABILITY: PROCEDURAL KNOWLEDGE**  **REFLECTIVE MOTIVATION: VALUES, INTENTIONS, SELF-IDENTITY**  **AUTOMATIC MOTIVATION: HABITS, TASTE, EMOTIONS**  **PHYSICAL CAPABILITY: SKILLS, FINANCIAL**  **PHYSICAL OPPORTUNITY: CONVENIENCE, HOUSEHOLD MEMBER COMMITMENTS, FOOD ENVIRONMENT** |

| **PART 7: TRIAL MAKING A MEDITERRANEAN or VEGETARIAN MEAL (10 MINUTES)** | |
| --- | --- |
| **Aim** | Get participants to cook one of the meals (if they are willing) and take a photo (All participants) |
| **Stimulus** | PowerPoint Slide with pictures of MEDITERRANEAN and VEGETARIAN MEALS |

| **RESPONSE TO MAKING A MEDITERRANEAN/ VEGETARIAN MEAL** | **COM-B Section & Observation notes** |
| --- | --- |
| ***Follow up phone call in 1 week***  *How did you go with cooking a Mediterranean or vegetarian meal? If no, thank them for their time, if yes, continue below:*  Which meal did you choose? How did it go?  PROMPT on if necessary:   - Affordability - Ingredients / availability - What was easy? - What was hard? - Did everyone eat it? Waste? - How did it taste/smell? - Did you feel full? - How did you feel? - Did you take a photo – can you send it to me? | **PSYCHOLOGICAL CAPABILITY: PROCEDURAL KNOWLEDGE**  **REFLECTIVE MOTIVATION: VALUES, INTENTIONS, SELF-IDENTITY**  **AUTOMATIC MOTIVATION: HABITS, TASTE, EMOTIONS**  **PHYSICAL CAPABILITY: SKILLS, FINANCIAL**  **PHYSICAL OPPORTUNITY: CONVENIENCE, HOUSEHOLD MEMBER COMMITMENTS, FOOD ENVIRONMENT** |
