# Supplementary material for: Barriers and drivers to adopting a plant-rich Mediterranean diet in a high-income country: A qualitative study
Source: J Health Psychol. 2025 Aug 3;31(4):1345–61. doi: 10.1177/13591053251354851 (PMC12960785; doi:10.1177/13591053251354851)
Supplement: sj-docx-2-hpq-10.1177_13591053251354851 – Supplemental material for Barriers and drivers to adopting a plant-rich Mediterranean diet in a high-income country: A qualitative study [file sj-docx-2-hpq-10.1177_13591053251354851.docx]

| **Participant Demographics and Diet Group** | | | | | | | | |
| --- | --- | --- | --- | --- | --- | --- | --- | --- |
| Pseudonym | Gender | Age | Heritage | Household Type | Income  ‘000 | Education Attainment | Current Diet | Likelihood of MD adoption  (0-10) |
| *Engaged* | | | | | | | | |
| Justin | Male | 24 | Australian | Couple | 170-179 | Tertiary | VEG | 1 |
| Greta | Female | 29 | European | Single parent with children | 20-29 | Tertiary | FLEX/SV | 8 |
| Leah | Female | 33 | Australian | Couple with children | 130-139 | Tertiary | MD | 3 |
| William | Male | 55 | Australian | Couple | 70-79 | TAFE | FLEX/SV | 6 |
| Shahen | Female | 64 | Assyrian | Extended Family | 60-69 | Tertiary | MD | 8 |
| *Likely to Adopt* | | | | | | | |  |
| Rebecca | Female | 35 | Australian | Couple with children | 170-179 | Tertiary | OMN | 7 |
| Kelly | Female | 37 | Australian | Couple | 180+ | Tertiary | OMN | 10 |
| Jessica | Female | 43 | Australian | Couple with children | 180+ | Tertiary | OMN | 9 |
| Brian | Male | 47 | Australian | Couple | 180+ | < High school | OMN | 8 |
| Laura | Female | 50 | Australian | Couple with children | 180+ | High school | OMN | 8 |
| Mark | Male | 65 | Australian | Couple | 110-120 | Tertiary | OMN | 9 |
| *Unlikely to Adopt* | | | | | | | |  |
| Raymond | Male | 28 | Australian | Couple | 80-89 | High school | OMN | 0 |
| Alison | Female | 34 | Australian | Extended Family | 100-109 | TAFE | OMN | 1 |
| Katherine | Female | 31 | Australian | Couple with children | 140-149 | Tertiary | OMN | 2 |
| Ryan | Male | 35 | Australian | Couple | 150-159 | High school | OMN | 4 |
| Martin | Male | 44 | Australian | Couple | 120-129 | TAFE | OMN | 5 |

OMN = Omnivore; MD = Mediterranean diet; FLEX/SV = flexitarian/semi-vegetarian; VEG = vegetarian.

Higher scores on the likelihood of adoption scale indicate greater likelihood of adoption.

To assess their current diet, participants viewed six diets—omnivore, MD, flexitarian/semi-vegetarian, pescatarian, vegetarian, and vegan—and selected the diet that most closely described what they had eaten over the last week. To assess their likelihood of adopting the MD, participants indicated this on an 11-point scale, where 0 = not at all likely, and 10 = extremely likely. Based on these responses, participants were placed into one of three groups: *Engaged* (i.e., currently consuming a MD or other plant-rich diet), *Likely to Adopt* (i.e., currently consuming an omnivore diet and a likelihood of MD adoption score of six or more), and *Unlikely to Adopt* (i.e., currently consuming an omnivore diet and a likelihood of MD adoption score of five or less).
