# Supplementary figures and images for: Barriers and drivers to adopting a plant-rich Mediterranean diet in a high-income country: A qualitative study
Source: J Health Psychol. 2025 Aug 3;31(4):1345–61. doi: 10.1177/13591053251354851 (PMC12960785; doi:10.1177/13591053251354851)

Mediterranean Diet Pyramid (Medical News Today)


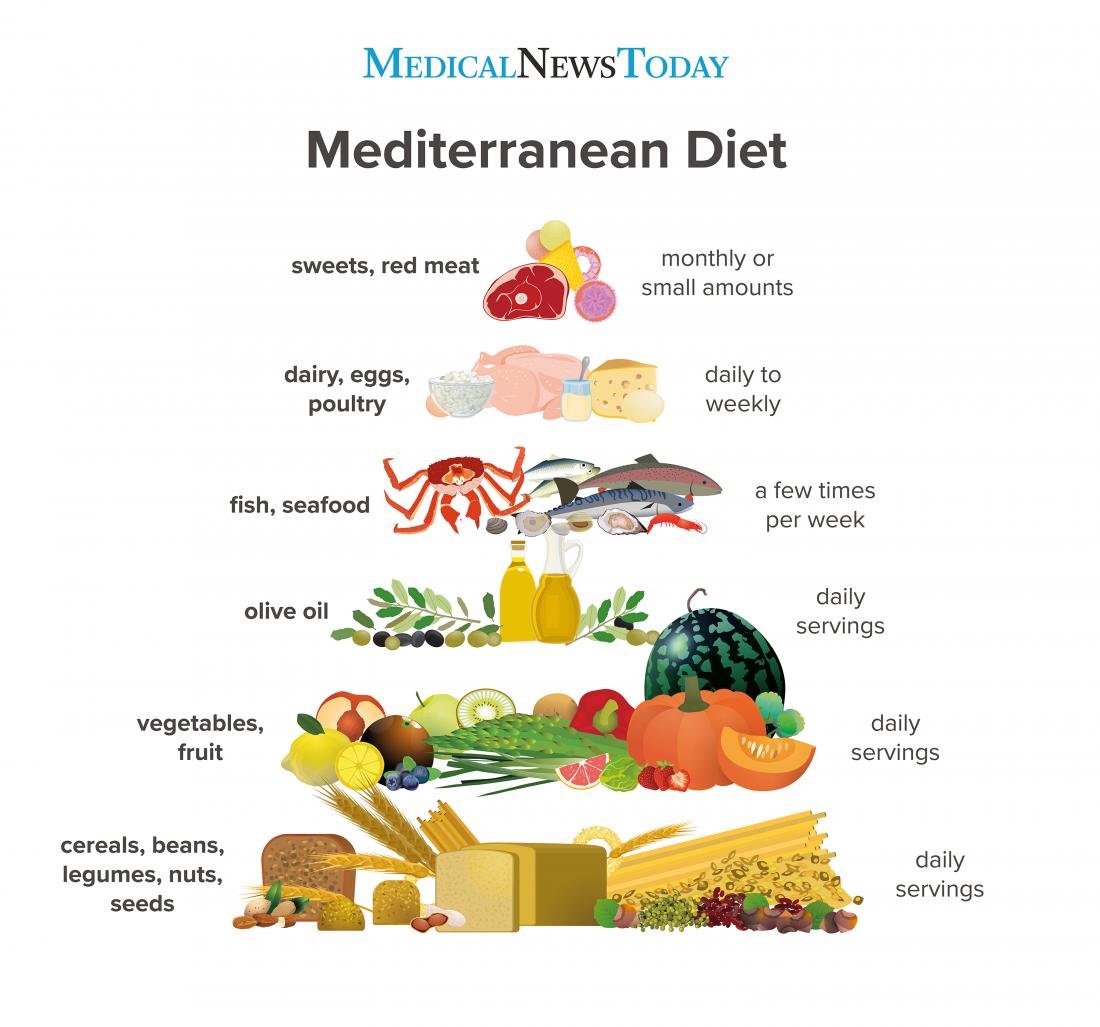


Mediterranean Diet Plate (Durrer Schutz et al., 2019)

Supplement: sj-docx-3-hpq-10.1177_13591053251354851 – Supplemental material for Barriers and drivers to adopting a plant-rich Mediterranean diet in a high-income country: A qualitative study [file sj-docx-3-hpq-10.1177_13591053251354851.docx]
